# Detection and quantification of human immunodeficiency virus-1 (HIV-1) total nucleic acids in wastewater settled solids from two California communities

**DOI:** 10.1128/aem.01477-24

**Published:** 2024-11-11

**Authors:** Marlene K. Wolfe, Meri R. J. Varkila, Alessandro Zulli, Julie Parsonnet, Alexandria B. Boehm

**Affiliations:** 1 Gangarosa Department of Environmental Health, Rollins School of Public Health, Emory University, Atlanta, Georgia, USA; 2 Division of Infectious Diseases and Geographic Medicine, Department of Medicine, Stanford University, Stanford, California, USA; 3 Department of Civil and Environmental Engineering, Stanford University, Stanford, California, USA; 4 Department of Epidemiology and Population Health, Stanford University, Stanford, California, USA; Centers for Disease Control and Prevention, Atlanta, Georgia, USA

**Keywords:** wastewater, HIV, public health

## Abstract

Wastewater surveillance for infectious agents has proved useful in identifying the circulation of viruses within populations. We investigated the presence and concentration of human immunodeficiency virus (HIV)-1 total nucleic acids (including both viral RNA and proviral DNA) in wastewater solids. We retrospectively measured HIV-1 nucleic acids in two samples per week for 26 months at two wastewater treatment plants serving populations with different prevalences of HIV infections in San Francisco and Santa Clara County, California, USA. We detected HIV nucleic acids in a majority of samples with concentrations ranging from non-detect to 3.9 × 10^5^ cp/g (*N* = 459 samples total). Concentrations of HIV-1 were significantly higher in samples from the wastewater treatment plant serving a population with a higher prevalence of people living with HIV than in the plant serving a population with a lower prevalence. The HIV-1 nucleic acids amplified were primarily DNA and thus represented proviral DNA shedding into wastewater. Additionally, we found that HIV-1 nucleic acid concentrations in wastewater solids were orders of magnitude higher than those in liquid wastewater indicating that the HIV-1 target preferentially sorbs to solids. Whether concentrations of HIV-1 in wastewater solids can be used to identify the number of incident cases remains unknown. Additional work on HIV-1 shedding from individuals with viremia and people living with HIV is needed to translate wastewater measurements into quantitative information on infections. Additional work may also be needed to document non-human sources of HIV-1 nucleic acids in wastewater.

**IMPORTANCE:**

Human immunodeficiency virus (HIV)-1 has infected nearly 100 million people since it emerged in the 1980s. Antiretroviral therapy prevents transmission of HIV and also allows infected individuals to live healthy lives with normal life expectancy. Consequently, identifying unrecognized cases of HIV is of paramount importance. Since wastewater represents a composite biological sample from a community, it may provide valuable information on HIV-1 prevalence that can be used to inform HIV testing and outreach.

## INTRODUCTION

Despite advances in prevention and treatment and a decrease in new human immunodeficiency virus (HIV) infections worldwide, more than 600,000 people die from acquired immunodeficiency syndrome annually (1). The Ending the HIV Epidemic in the United States (EHE) initiative has set an ambitious goal of reducing the number of new HIV infections in the US by 90% between 2019 and 2030 (2). Progress, however, has been below target and disproportionately slow in some demographic groups and geographic regions. At the end of 2021, an estimated 1.2 million people were living with HIV (PLWH) in the US, and 32,700 people were newly diagnosed (3).

Minoritized people are disproportionately affected by the HIV epidemic in the US; 39% of people receiving a new diagnosis in the US in 2023 were Black (4). Although it has been estimated that 87% of those living with HIV in the US are aware of their status, the CDC has estimated that in 2022, 43% of PLWH remained viremic. During the coronavirus disease 2019 pandemic, HIV testing and new diagnoses decreased, making it possible that fewer PLWH are aware of their status and receiving treatment (5). Systemic barriers to care, exacerbated by the pandemic, may continue to slow progress toward EHE goals. Ongoing assessment of the prevalence of HIV infection in communities, especially those underserved by healthcare and testing centers, is critical to provide resources that ensure consistent testing and subsequent treatment.

Wastewater monitoring has not been previously applied to HIV but could help identify trends in infection prevalence and identify pockets of underdiagnosed infection. Wastewater, a naturally composited biological sample that includes bodily secretions, has been used to monitor a wide range of infectious diseases including respiratory viruses, enteric viruses, dengue (an arbovirus), and mpox (a poxvirus) (6–12). Wastewater monitoring was effectively used to support mpox response at the start of the outbreak in the US during the summer of 2022 (8), providing information to respond to an outbreak that was syndemic (13) with HIV (14).

Although the use of wastewater monitoring for HIV has not been explored, evidence from early in the HIV pandemic suggests that viral RNA and DNA can be detected in wastewater. Several studies from the early 1990s demonstrated the detection of HIV nucleic acids (NA) and intact viruses in natural wastewater (15, 16) and the detectability of the virus seeded in wastewater (17, 18). These studies were focused on occupational hazards, rather than monitoring. Some data indicate that HIV nucleic acids are shed in the urine and feces of PLWH, even those who are virally suppressed (19–21). Contributions to wastewater therefore may be expected from PLWH. This suggests that detection and quantification for monitoring of HIV infections at the community level is a realistic possibility.

The goal of this study is to apply testing for HIV-1 RNA and DNA to wastewater samples from two adjacent counties to determine whether consistent detection and quantification of HIV-1 nucleic acids in wastewater is feasible and to explore the relationship of these measurements to the overall estimated rates of people living with HIV in the associated communities. Although HIV can be caused by both HIV-1 and HIV-2, this study focuses on HIV-1 (>99.9% of HIV cases in the US).

## MATERIALS AND METHODS

### Study design and sample collection

We conducted a longitudinal retrospective analysis of HIV nucleic acids in wastewater solids from two wastewater treatment plants (WWTPs) in the San Francisco Bay Area, California, USA. Fifty milliliters of wastewater solids was collected from primary clarifiers daily using sterile methods and biobanked with the consent of the participating WWTPs as part of a routine wastewater monitoring program that began in November 2020. Two samples per week covering a period of 26 months were selected for this study. They were collected between 2 February 2021 and 14 April 2023 (459 samples total). The two WWTPs included in the retrospective analysis serve ~75% (1,500,000 people) of Santa Clara County (SJ) and ~25% (250,000 people) of San Francisco County (OSP), CA. Further WWTP descriptions are elsewhere (22). Samples were stored at 4°C, transported to the lab, and processed within 6 h. Solids were then dewatered using centrifugation (23) and then biobanked by freezing at −80°C for 4–60 weeks until further analysis.

We collected additional new samples over a consecutive 9 days to compare concentrations of HIV-1 between liquid wastewater and wastewater solids and to compare measurements made with and without a reverse transcription step during the analytic analysis. Additional samples were collected from two sites, OSP and a second site in San Francisco County (SEP) that serves ~75% of the county (650,000 people). For these two sites, 24 h, flowtime-weighted (OSP) and 24 h timed (SEP, one sample every 30 min) composite samples of raw wastewater influent (liquid) and a grab sample of solids from the primary clarifiers were collected on the same day for 8 days in early July 2023, and one additional solids sample was collected from SEP (Table S1, samples hereafter labeled 1–8 for OSP and SEP liquids, and 1–8 and 1–9 for OSP and SEP solids, respectively). The autosamplers used for the influent sampling were Teledyne ISCO 5800 samplers (Lincoln, NE, USA). All the solids samples (*n* = 17) were tested for HIV-1 target using both reverse transcription-PCR (RT-PCR) and PCR to gain insight into the proportion of HIV-1 RNA and DNA in the samples, respectively (Table S1). Samples were stored at 4°C for the duration of the 9-day study and then underwent pre-analytical and analytical processing without any additional storage. Solids were dewatered immediately using centrifugation (23) and subsequently processed for nucleic acid extraction without storage.

### Solids pre-analytical methods

If frozen, dewatered solids were thawed overnight at 4°C. Solids were resuspended in DNA/RNA Shield (Zymo Research, Irvine, CA, USA) at a concentration of approximately 0.75 mg (wet weight)/mL. This concentration was chosen as it was shown to reduce inhibition in RT-PCR applications with extracted nucleic acids (24). A separate aliquot of dewatered solids was dried in an oven to determine its dry weight, and the calculated percent solids was used to express concentrations of nucleic acid targets in units of per gram dry weight. These pre-analytical methods are described thoroughly in a publicly accessible protocol (25). Nucleic acids were extracted from 10 replicate aliquots of dewatered settled solids suspended in the DNA/RNA Shield using the Chemagic Viral DNA/RNA 300 Kit H96 for the Perkin Elmer Chemagic 360 (PerkinElmer, Waltham, MA, USA) followed by PCR inhibitor removal with the Zymo OneStep-96 PCR Inhibitor Removal Kit (Zymo Research, Irvine, CA, USA). Three hundred microliters of the suspension entered into the nucleic acid extraction process and 50 µL of nucleic acids are retrieved after the inhibitor removal kit. Negative extraction controls consisted of DNA/RNA Shield that went through the extraction procedure. Nucleic acid extracts used in the retrospective study were stored at −80°C for different amounts of time (8–273 days, median = 266 days) and subjected to one freeze thaw prior to analysis. Nucleic acids from the solids samples used for the liquid/solid comparison and with/without reverse transcription were not stored prior to analysis.

### Liquids pre-analytical methods

Viral particles were concentrated from wastewater using an affinity‐based capture method with magnetic hydrogel Nanotrap Particles A (Ceres Nanosciences, Manassas, VA, USA). For each sample, 10 replicate aliquots of 10 mL of liquid wastewater were concentrated using the Nanotrap Particles and Enhancement Reagent 1 on a KingFisher Flex system. Nucleic acids were then extracted from each replicate with the MagMAX Viral/Pathogen Nucleic Acid Isolation Kit (Applied Biosystems, Waltham, MA, USA) using the KingFisher Flex system. Resulting nucleic acids were then processed through a Zymo OneStep-96 PCR Inhibitor Removal Kit (Zymo Research, Irvine, CA, USA). Each 10 mL sample resulted in 50 µL total nucleic acid extract. Note that suspended solids were not removed before initiating these methods, and the nucleic acids were not stored prior to analysis.

### HIV (reverse transcription)-PCR assay choice

We selected previously published HIV genome-specific primers and probes targeting the long terminal repeat (LTR) region of the HIV-1 genome for use in this study; the assay chosen does not target HIV-2 (26). We used the 496F/546P/622R primers and probe as recommended by Kibirige et al. (27). The assay uses primers and probes originally described in Brussel et al. (28) and Friedrich et al. (29) (Table 1).

**TABLE 1:** Primer and hydrolysis probes targeting the LTR region of HIV-1^*a*^

	Sequence
Forward	GGCTAACTAGGGAACCCACTG
Reverse	TCCACACTGACTAAAAGGGTCTGA
Probe	CACTCAAGGCAAGCTTTATTGAGGC

^*a*^ The amplicon size is 127 bp. The HIV-1 probe contained Cyanine-5 (Cy5) fluorescent molecule; ZEN, a proprietary internal quencher from Integrated DNA Technologies (Coralville, IA, USA); and IBFQ, Iowa Black FQ.

To confirm HIV-1 assay specificity, the assay was challenged with non-target viruses including those in two panels purchased from Zeptomatrix (Buffalo, NY, USA), NATRVP2.1-BIO, and NATEVP-C, along with HIV-2 (ATCC PTA-9773). The NATRVP2.1-BIO panel includes chemically inactivated intact influenza viruses, parainfluenza viruses, adenovirus, rhinovirus, metapneumovirus, and coronaviruses. The NATEVP-C panel includes chemically inactivated intact coxsackieviruses, echovirus, and parechovirus. To confirm HIV-1 assay sensitivity, synthetic HIV-1 RNA (ATCC VR-3351SD) was used as a positive control for HIV-1. American Type Culture Collection (ATCC) controls are synthetic RNA. NA were extracted from intact viruses using the Chemagic Viral DNA/RNA 300 Kit H96 (PerkinElmer, Waltham, MA, USA).

### Droplet digital RT-PCR methods

For sensitivity and specificity testing of the HIV-1 assay, nucleic acids were used undiluted as templates in digital RT-PCR reactions in a single well. No template negative RT-PCR controls using molecular grade water as the template were included in each plate. The assay was run in simplex with only primers and probe listed in Table 1.

Wastewater samples were tested for HIV-1 nucleic acids as well as pepper mild mottle virus (PMMoV) using droplet digital RT-PCR. PMMoV is a pepper virus that is highly abundant in wastewater across the world and is used in this study as an endogenous recovery control. We expect to detect it in relatively high concentrations given our experience with this virus in wastewater (24), and it acts as a measure of fecal strength of the wastewater (30). We expect to detect PMMoV in relatively high concentrations in all wastewater samples (27). We did not utilize an exogenous extraction control (such as a spiked virus-like bovine coronavirus) due to the difficulty in interpreting recovery with an exogenous target (31); however, the described pre-analytical methods have consistently provided high recoveries approaching 100% of spiked BCoV in our laboratories (22). We acknowledge that quantifying recovery of nucleic acids in samples, using both endogenous and exogenous controls, is challenging, as described by Kantor et al. (31).

Each of the 10 replicate nucleic acid wastewater extracts was used as template undiluted in its own well (10 wells per sample) to measure HIV-1 and PMMoV. The HIV-1 assay was run in multiplex using a probe mixing approach with seven other assays targeting genomes of rotavirus, influenza A subtype markers H1 and N1, severe acute respiratory syndrome coronavirus 2 (SARS-CoV-2), human norovirus GII, human adenovirus group F, and West Nile virus [results not reported herein, but provided elsewhere (32, 33)]. Multiplexing method testing showed that the presence of seven other nucleic acid targets in the multiplex reaction did not affect quantification of the HIV-1 target (Fig. S1). Nucleic acids were diluted 1:100 and used as a template to measure PMMoV using methods outlined in detail elsewhere (22) and thoroughly in a protocols.io protocol (34) (Table S2 provides primers and probe). Extraction negative (DNA/RNA Shield, three wells) controls and PCR negative (molecular grade water, three wells) and positive controls (synthetic RNA, one well) were run on each 96-well plate. Any failure of these controls would result in re-processing of the samples, and any samples with anomalously low PMMoV would also be rerun or discarded from the analysis.

Droplet digital RT-PCR was performed on 20 µL samples from a 22-µL reaction volume, prepared using 5.5 µL template, mixed with 5.5 µL of One-Step RT-ddPCR Advanced Kit for Probes (Bio-Rad 1863021), 2.2 µL of 200 U/µL reverse transcriptase, 1.1 µL of 300 mM dithiothreitol (DTT) and the remaining volume consisting of water and primers and probes mixtures at a final concentration of 900 nM and 250 nM, respectively. Twenty-two microliters is prepared since the droplet generator leaves 2 µL behind during droplet generation. Primers and probes for assays were purchased from Integrated DNA Technologies (San Diego, CA, USA) (Table 1; Table S2).

HIV-1 nucleic acids were quantified in the 17 solids samples collected at OSP and SEP in July 2023 (Table S1) using both droplet digital RT-PCR and droplet digital PCR without the RT step. Droplet digital RT-PCR was run as described above. Droplet digital PCR was run using the same method as for droplet digital RT-PCR but the RT enzyme and DTT were omitted from the mastermix. As a control, the concentration of the N gene of SARS-CoV-2 was examined from the multiplex assay as the N gene is expected to only be detected when an RT step is included as it resides in wastewater as genomic RNA. We confirmed that the N gene was not detected without the RT step indicating lack of RT activity in that mastermix.

Droplets were generated using the AutoDG Automated Droplet Generator (Bio-Rad, Hercules, CA, USA). PCR was performed using Mastercycler Pro (Eppendorf, Enfield, CT, USA) with the following cycling conditions: reverse transcription at 50°C for 60 min, enzyme activation at 95°C for 5 min, 40 cycles of denaturation at 95°C for 30 s, and annealing and extension at 59°C (for HIV-1) or 56°C (for PMMoV) for 30 s, enzyme deactivation at 98°C for 10 min then an indefinite hold at 4°C. The ramp rate for temperature changes was set to 2°C/s, and the final hold at 4°C was performed for a minimum of 30 min to allow the droplets to stabilize. Droplets were analyzed using the QX200 or the QX600 Droplet Reader (Bio-Rad). A well had to have over 10,000 droplets for inclusion in the analysis. All liquid transfers were performed using the Agilent Bravo (Agilent Technologies, Santa Clara, CA, USA).

Thresholding was done using QuantaSoft Analysis Pro Software (Bio-Rad, version 1.0.596) for data generated using the QX200 and QX Manager Software (Bio-Rad, version 2.0) for data generated using the QX600. In order for a sample to be recorded as positive, it had to have at least three positive droplets across merged wells. Replicate wells were merged for analysis of each sample which essentially increases the volume of template. Equivalent sample mass or volume for the solids and liquid samples across 10 merged wells is estimated at 0.02 g wet weight and 11 mL, respectively.

Dimensional analysis was used to convert concentrations of nucleic acid targets from copies per reaction to copies per dry weight of solids (cp/g) or copies per milliliter (cp/mL) of influent. Measurement errors are reported as standard deviations. The lowest detectable concentrations were ~500–1,000 cp/g for solids and ~1 cp/mL for liquids, respectively, and is equivalent to the concentration giving rise to three positive droplets across 10 merged wells. The range provided for solids reflects differences in the dry weight of different solids samples. Wastewater data for the retrospective study are publicly available through the Stanford Digital Repository (https://doi.org/10.25740/yz257qj0009).

### Statistics

A Wilcoxon rank sum test was used to test the null hypothesis that there was no difference in the concentrations of nucleic acids at the two wastewater treatment plants with the alternative hypothesis that concentrations were higher at OSP than SJ. A Wilcoxon signed rank test was used to test the null hypothesis that there was no significant difference in the concentration between matched samples tested with and without a reverse transcription step. A Wilcoxon signed rank test was also used to test the null hypothesis that there was no significant difference in the concentration estimated on a per-mass basis (cp/g and cp/mL) between matched liquid and solids samples.

### Clinical data

Estimated annual numbers of incident cases of HIV were obtained at the county level from publicly available county health department surveillance summaries for 2021 and 2022 (35–38). Estimates of PLWH and the proportions with viral suppression were also obtained from these reports. Data for 2023 were not available at the time of writing; further details of health department reporting and definitions are available in the SI. Rates of PLWH per county were calculated based on yearly population denominators obtained from the State of California, Department of Finance, Demographic Research Unit (39).

## RESULTS

### Quality control

Positive and negative extraction and PCR controls were positive and negative, respectively, indicating assays performed and no evidence of contamination. The PMMoV endogenous control, as expected, was detected at relatively high concentrations in wastewater at the two sites [median (range) log_10_-transformed PMMoV = 9.1 (8.5–10.2) and 8.7 (8.1–9.5) log_10_ copies/g at SJ and OSP, respectively]. Given that the lowest measurements at the two sites were within an order of magnitude of the site medians, we concluded that there was no gross extraction failure. No samples were identified as needing to be rerun due to quality control failures. Further details of the methods and controls per the Environmental Microbiology Minimal Information guidelines can be found in the Supplementary Materials (Fig. S2).

HIV-1 assay sensitivity and specificity were previously established by researchers who developed and recommended the HIV-1 assay, and our *in vitro* testing herein confirmed assay performance; there was no observed cross reactivity, and the assay detected HIV-1 at the lowest challenged concentration of 3 copies per reaction (Fig. S1).

We compared PMMoV measured using the biobanked samples (reported in this study using the methods described in the Materials and Methods) to PMMoV measured in the same samples when processed immediately for a routine wastewater surveillance project; the results from the samples analyzed without any storage are reported by Boehm et al. (24). Median (interquartile range) ratio of PMMoV RNA measurements made in this study to those made using fresh samples was 0.9 (0.6–1.2) at SJ and 1.0 (0.7–1.4) at OSP suggesting limited degradation of the PMMoV RNA target in the biobanked samples (which were stored and then freeze thawed for use in the study). It should be noted that there could have still been degradation of HIV-1 nucleic acids in these biobanked samples, especially since the N gene from SARS-CoV-2 has shown some losses under these storage conditions (40); however, sample storage is essential for retrospective work.

### Analysis of HIV-1 in wastewater

**Fig 1:**
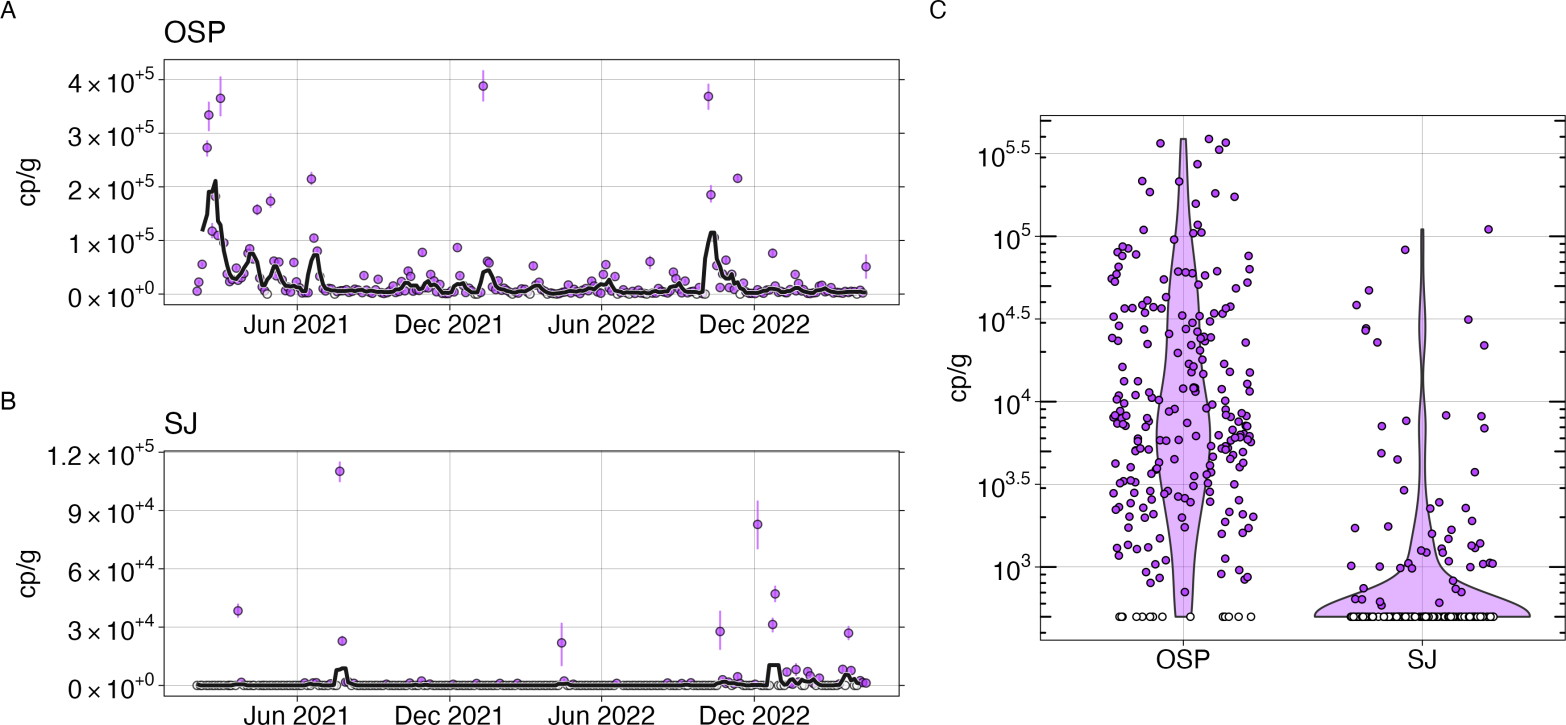
Wastewater concentrations of HIV nucleic acids. Left: concentration of HIV nucleic acids in wastewater solids from OSP (**A**) and SJ (**B**) expressed in terms of copies of the target per gram dry weight of solids found in wastewater. Open circles show where HIV nucleic acids were not detected, and the errors on each point represent standard deviations from the droplet digital RT-PCR measurement and include Poisson error as well as variation among replicate wells. The black line represents the 5-sample smoothed and trimmed average. Right: distribution of the concentration of HIV nucleic acids in wastewater solids from OSP and SJ (**C**), in terms of copies of the target per gram dry weight of solids found in wastewater. Open circles show where HIV nucleic acids were not detected; samples in which HIV NA were not detected were assigned a value of 500 cp/g to represent the estimated lower limit of detection. The shaded region is a violin plot, and the width of each shape represents the frequency of data points at different concentrations.

HIV-1 NA were detected consistently throughout the retrospective study period in both OSP and SJ wastewater (Fig. 1). HIV-1 NA were detected in 94% (215/230) of samples from OSP and 23% (53/229) of samples from SJ. Measured HIV-1 NA concentrations were significantly higher at OSP compared to SJ (Wilcoxon rank sum test, *P* < 0.001, Fig. 1). At OSP, concentrations ranged from non-detect to 3.9 × 10^5^ cp/g with a median of 7.3 × 10^3^ cp/g. At SJ, concentrations ranged from non-detect to 1.1 × 10^5^ cp/g with a median of non-detect.

**Fig 2:**
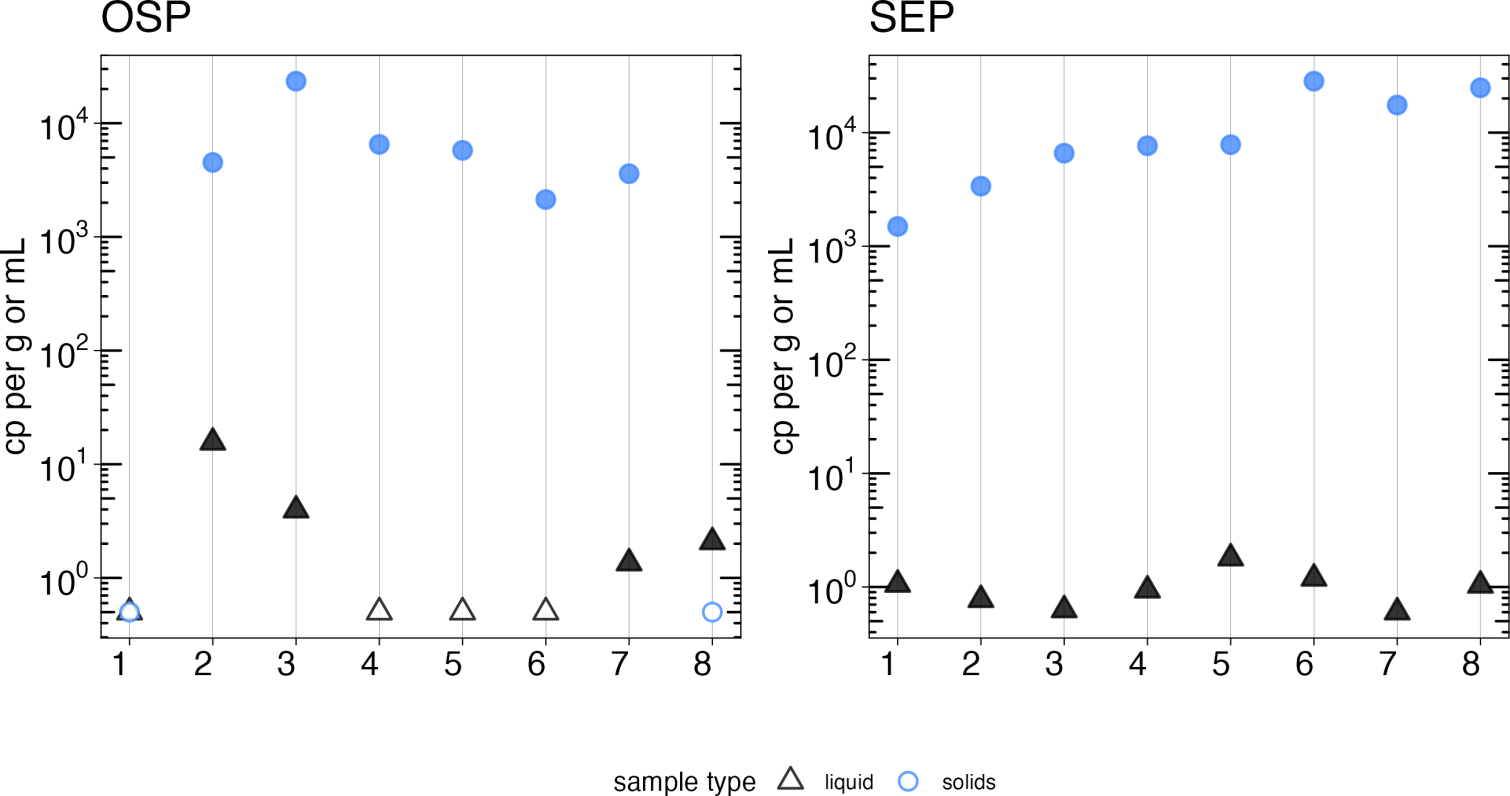
Comparison of HIV nucleic acid quantification in liquids and solids. Concentrations of HIV nucleic acids in wastewater liquids (black triangles) and solids (blue circles) from OSP and SEP expressed in terms of copies of the target per milliliter of liquid wastewater or grams dry weight of solids found in wastewater. Open circles or triangles show where HIV nucleic acids were not detected.

Measured HIV-1 NA concentrations were significantly different in solids compared to liquids (Wilcoxon signed rank test, *P* < 10^−3^). Concentrations in solids were enriched by several orders of magnitude compared to liquids on a per-mass basis with a median concentration of 1 cp/mL in liquids and 6,142 cp/g in solids across all samples included in the comparison (Fig. 2). Liquid samples also had a higher rate of non-detection for HIV-1; HIV-1 NA were not detected in 4/16 liquids samples and 2/16 solids samples. One sample had no HIV-1 NA detected by either method.

**Fig 3:**
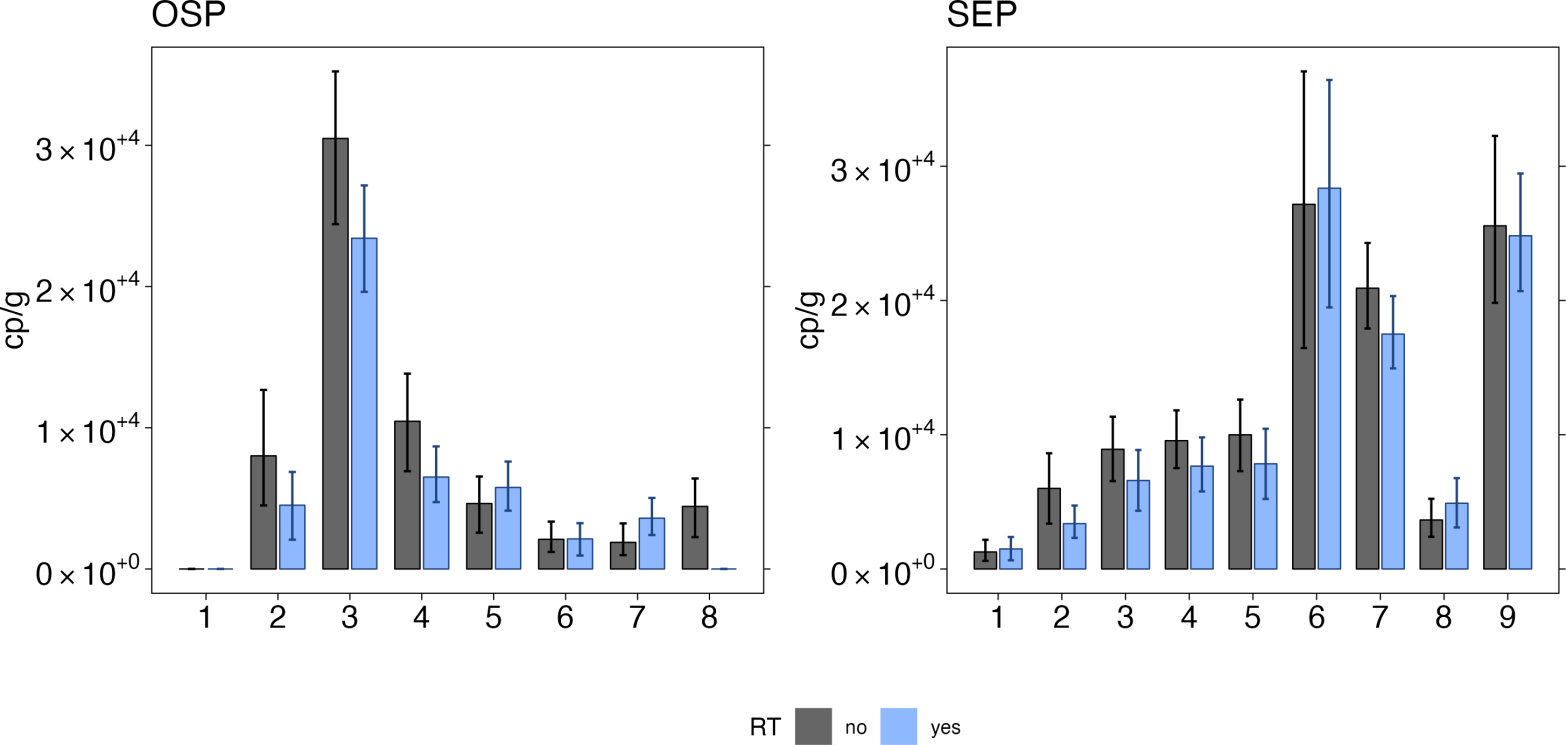
Comparison of HIV nucleic acid quantification with and without RT. Concentrations of HIV nucleic acids in wastewater solids from OSP and SEP are expressed in terms of copies of the target per gram dry weight of solids found in wastewater for quantification with the use of RT (blue) and without (black). Errors on each bar represent standard deviations from the droplet digital RT-PCR measurement and include Poisson error as well as variation among replicate wells.

When analyzed with and without a reverse transcription step, HIV-1 NA in wastewater were significantly different (Wilcoxon signed rank test, *P* = 0.03) but the magnitude of the difference between the two was small (Fig. 3; with RT median = 5,773 cp/g and mean = 8,734 cp/g, without RT median = 8,012 cp/g and mean = 10,296 cp/g).

### Clinical data

The number of new incident HIV infections in San Francisco was similar to the number in Santa Clara County for both 2021 and 2022 (Table 2); however, as the population of Santa Clara County is much larger, the rate of new diagnoses per 100,000 residents was higher in SF than in SCC. The case counts and prevalences of PLWH were also higher in San Francisco County compared to Santa Clara County. The two counties have similar viral suppression proportions, estimated at 72%–73% for San Francisco and 70.2%–68.1% for Santa Clara County from 2021 to 2022.

**TABLE 2:** Numbers and rates of PLWH, %PLWH who were virally suppressed, and new HIV diagnoses in San Francisco and Santa Clara counties in 2021 and 2022 (35–38)

	San Francisco County		Santa Clara County	
	2021	2022	2021	2022
Total PLWH, *N*	11,830	11,798	3,618	3,770
PLWH per 100,000 population^*a*^	1,405.5	1,399.4	190.4	198.1
New diagnoses, *N*	166	157	133	165
New diagnoses per 100,000 population^*a*^	19.7	18.6	7.0	8.7
Viral suppression, %	72	73	70.2	68.1

^*a*^ Rates per 100,000 population are based on population data from 2023 from the State of California (41).

These county level HIV prevalence estimates serve as imperfect proxies for the PLWH in the OSP and SJ sewersheds. OSP WWTP serves 25% of the population of San Francisco County, and annual reports show that the geographic area served by the plant has lower rates of PLWH than the county as a whole (38, 42). Neighborhoods within the OSP service area are described as having a prevalence of 317–999/100,000 PLWH in 2021 and 310–799/100,000 PLWH in 2022. SJ WWTP serves 75% of the population of Santa Clara County; it is not publicly available how PLWH are distributed in the county.

### Comparison of clinical and wastewater data

Overall, the rate of detection and concentrations of HIV-1 NA were higher in San Francisco County than in Santa Clara County, and new HIV diagnoses and the prevalence of PLWH were also higher in San Francisco County. No statistics are applied to this comparison as clinical data are available at an annual scale and *N* = 4, limiting appropriate hypothesis testing.

## DISCUSSION

This study demonstrates a first proof of concept that HIV-1 nucleic acids can be reliably detected and quantified in wastewater, and rates of detection and concentration are aligned with known information about the overall prevalence of PLWH in the associated communities. HIV-1 nucleic acids were consistently detected in wastewater from WWTPs in the San Francisco Bay Area, California, USA, with differences in wastewater rates of detection and concentrations that mirrored clinical surveillance data between counties. Both the rate of detection and concentrations of HIV-1 nucleic acids were higher in San Francisco County than in Santa Clara County. These findings are consistent with the higher prevalence of PLWH and the greater number of incident HIV cases in San Francisco. Although the sewershed area served by the San Francisco County WWTP covers a part of the city that has a relatively lower prevalence of residents with PLWH, even these areas have reported a prevalence that is approximately double or more the prevalence in Santa Clara County.

HIV-1 nucleic acids were detected from both the liquid and solid portions of wastewater from two sites in San Francisco County. The concentrations in solids were several orders of magnitude higher on a per-mass basis than those in liquid, and the rate of non-detection was slightly higher in liquid samples. This suggests that HIV-1 nucleic acids can be successfully quantified in wastewater utilizing either the solid or the liquid matrix, but the use of solids may improve the sensitivity of the measurements. Future work utilizing liquids and solids across a wider range of anticipated concentrations of HIV-1 NA would help determine the performance of various methods utilizing each matrix at different levels.

The field of wastewater monitoring for infectious diseases has grown rapidly in recent years and has not only become a part of many public health department’s approaches to infectious disease surveillance, and it has been successfully used to monitor diseases like mpox that interact with HIV (considered syndemics) (14, 43). Wastewater monitoring has not, however, been previously used to support HIV monitoring. This is likely because HIV has a number of important differences from other viruses commonly monitored using wastewater, such as respiratory and enteric viruses, particularly when considering the possibility of long-term shedding and how this relates to individuals with different disease outcomes (19, 20).

There are limited quantitative data on the concentration of HIV-1 nucleic acids in human excretions that are shed via drains that convey wastewater to treatment plants including urine and feces. While individuals with active infections may shed viral RNA, PLWH who are virally suppressed may still excrete HIV-1 DNA (19). In this study, we quantified total HIV-1 nucleic acids which include both RNA and DNA; the methods we used cannot distinguish between the two. The overall similarity in concentrations of HIV nucleic acids with and without reverse transcription in a small subset of samples suggests that the majority of the nucleic acids detected in wastewater are DNA. This result may suggest that in this study, we primarily detected DNA from CD4 T-cells that were shed in stool (44). Further work will need to be done to better understand the shedding dynamics of HIV-1 nucleic acids in PLWH with and without viral suppression to provide a foundation to further interpret these results.

This work presents a proof of concept. In order to make HIV monitoring in wastewater a tool that is useful for public health response to the HIV epidemic, more work is necessary to understand the relationship between these measurements and PLWH and even more importantly, the people living with undiagnosed or unmanaged HIV in the community. Identifying the latter may help direct resources to communities in need. Sequencing or detecting specific segments of the HIV-1 genomic targets in wastewater related to drug resistance may help inform initial antiretroviral therapy choices (e.g., is the virus mutating, is pre-exposure prophylaxis contributing to resistance, etc.) without identifying individuals.

Despite CDC recommendations for routine HIV screening in healthcare settings since 2006 (45), HIV testing rates during ambulatory care visits have remained low (46, 47). With barriers to implementation ranging from provider perceptions of low HIV risk to institutional concerns surrounding the allocation of limited resources (46, 47), recent initiatives, such as the Ending the HIV Epidemic, propose refocused efforts to target areas with the highest burden of HIV (48, 49). More information on HIV NA shedding will be important to help better understand the relationship between wastewater HIV concentrations and infection and disease outcomes. Eventually, data on HIV from wastewater may be useful both at a facility and community level for monitoring trends to help inform community-focused screening efforts. Tools like this would provide opportunities to compare the burden of HIV across geographic areas including those where resources are limited, to provide information on the scope of the HIV burden in institutions like hospitals and correctional facilities, and possibly to determine patterns in resistance. This may be used to determine times and places when increased screening at sites may be useful, feeding into existing frameworks that identify priority areas for intervention.

There are several limitations to this study that are important to highlight. We did not attempt to sequence HIV-1 amplicons generated from wastewater solids. Amplicons were generated in droplets and not easily accessible for downstream sequencing. Sequencing of amplicons could yield information about HIV-1 diversity; however, it should be noted that the targeted region of the HIV-1 genome is highly conserved (hence, making it a good target for HIV-1 detection in wastewater). In addition, sequencing could confirm the amplification of the intended target, although the use of the internal probe should be highly effective at confirming the amplification and subsequent detection of the target HIV-1 sequence. An additional limitation is that, like any study that detects nucleic acids in wastewater, it is difficult to prove the measurements are detecting HIV-1 DNA or RNA shed by humans as opposed to synthetic nucleic acids used in experiments. However, synthetic nucleic acids including gene blocks, plasmids, or lentiviral vectors potentially containing the HIV-1 sequence used as a target in this study are not to be disposed of down a drain per biosafety guidelines. Additionally, according to a recent study by Harrison et al. (50), naked ssRNA or dsDNA nucleic acids are particularly labile (half-lives less than 20 min) in wastewater; the study did not look at the persistence of plasmids. The potential contribution of research-related vectors (like plasmids, PCR amplicons, or lentiviruses) to the nucleic acid signal observed in waste streams is an area where additional research is needed. Additional work detecting and sequencing different regions of the HIV-1 genome in wastewater could provide useful information on HIV-1 drug resistance and provide additional confirmation of human-shed HIV-1 presence and concentrations in wastewater. Finally, further work to document the presence of both RNA and DNA from HIV-1 would provide additional insight into the nature of the contributions to wastewater.

## SUPPLEMENTAL MATERIAL

Supplemental material aem.01477-24-s0001.docx Supplemental methods, Fig. S1 and S2, and Tables S1 to S3.

ASM does not own the copyrights to Supplemental Material that may be linked to, or accessed through, an article. The authors have granted ASM a non-exclusive, world-wide license to publish the Supplemental Material files. Please contact the corresponding author directly for reuse.
